# T Cells and CDDO-Me Attenuate Immunosuppressive Activation of Human Melanoma-Conditioned Macrophages

**DOI:** 10.3389/fimmu.2022.768753

**Published:** 2022-02-21

**Authors:** Gretel M. Torres, Heetaek Yang, Chanhyuk Park, Paul A. Spezza, Nikhil Khatwani, Rajan Bhandari, Karen T. Liby, Patricia A. Pioli

**Affiliations:** ^1^Department of Microbiology and Immunology, Geisel School of Medicine at Dartmouth, Lebanon, NH, United States; ^2^Department of Pharmacology and Toxicology, Michigan State University, East Lansing, MI, United States

**Keywords:** human, melanoma, macrophage, T cells, CDDO-Me

## Abstract

Melanoma tumors are highly immunogenic, making them an attractive target for immunotherapy. However, many patients do not mount robust clinical responses to targeted therapies, which is attributable, at least in part, to suppression of immune responses by tumor-associated macrophages (TAMs) in the tumor microenvironment (TME). Using a human *in vitro* tri-culture system of macrophages with activated autologous T cells and BRAF^V600E^ mutant melanoma cells, we now show that activated T cells and the synthetic triterpenoid the methyl ester of 2-cyano-3,12-dioxooleana-1,9(11)-dien-28-oic acid (CDDO-Me) attenuate immune suppression. Surface expression of CD206, CD16 and CD163 on melanoma-conditioned macrophages was inhibited by the addition of T cells, suggesting relief of immuno-suppressive macrophage activation. We also demonstrated that addition of CDDO-Me to tri-cultures enhanced T cell-mediated reductions in CCL2, VEGF and IL-6 production in a contact-independent manner. Because these results suggest CDDO-Me alters melanoma-conditioned macrophage activation, we interrogated CDDO-Me-mediated changes in macrophage signaling pathway activation. Our results indicated that CDDO-Me inhibited phosphorylation of STAT3, a known inducer of TAM activation. Collectively, our studies suggest that activated T cells and CDDO-Me synergistically relieve immune suppression in melanoma cultures and implicate the potential utility of CDDO-Me in the treatment of melanoma.

## Introduction

Although melanomas account for 4% of all skin cancers, they are responsible for 75-80% of skin cancer-related deaths (1, 2). Mounting evidence suggests that these tumors develop and progress because of both the host immune response and inflammatory cells within the TME. As mediators of inflammation, tumor-associated macrophages (TAMs) constitute a significant source of immune-suppression in the tumor microenvironment (TME). In this regard, TAMs induce regulatory T cell (Treg) generation, programmed death 1 (PD1)-dependent lymphocyte immunosuppression, and tumor-associated neo-angiogenesis (3–5). In addition, when murine myeloid derived suppressor cells (MDSCs) populate the TME, they become F4/80^+^ (6), suggesting MDSCS may constitute an important subpopulation of immuno-suppressive TAMs in malignant melanomas. Thus, interventions that redirect TAM activation from immuno-suppressive to immuno-stimulatory may have significant therapeutic benefit.

In this regard, T helper 1 (Th1) responses have been shown to promote immunostimulatory differentiation of TAMs in early tumors (7), and TAMs isolated from murine myelomas activate CD4^+^ T cells upon MHC-II engagement (8). These data suggest that T cells provide key signals required for TAM reprogramming and re-education. While many model systems address the role of macrophages in tumor eradication, few models include multiple immune cell types in the TME. Given the dynamic interaction between TAMs and T cells in the TME, it is imperative to consider the mutual influence of these immune cells on one another in the design of efficient therapeutics.

Triterpenoids are widely used in traditional Asian medicine and include oleanolic acid (OA), which has anti-inflammatory and anti-carcinogenic properties (9). The triterpenoid 2-cyano-3,12-dioxooleana-1,9-dien-28-oic acid (CDDO-Me) is a synthetic analog of OA that has anti-tumorigenic activity against a broad variety of cancer types *in vitro* and *in vivo* (10–12). Notably, CDDO-Me inhibits myeloid cell tumor infiltration and neutralizes immune suppression mediated by MDSCs through inhibition of ROS and IFN-γ T cell production (13). In recent studies, we have shown that CDDO-Me remodels the breast tumor TME, redirecting TAM activation from immunosuppressive to immunostimulatory (10). Our work also demonstrates that CDDO-Me alters the tumor T cell compartment by increasing the ratio of CD8 to CD4 T cells and by reducing Treg tumor infiltration (10).

In Phase I clinical trial testing of patients with advanced solid tumors and lymphomas, CDDO-Me was well-tolerated and mediated anti-cancer activity (14). Intriguingly, a third of the melanoma patients treated with CDDO-Me in this trial showed disease stabilization for 4-10 months (14). Given this observation and our published studies, we hypothesized that one mechanism by which CDDO-Me inhibits melanoma growth is through remodeling of the TME. To model the TME using human cells, we adopted a tri-culture system consisting of malignant melanoma cells, T cells, and macrophages. We established tri-cultures using the clinically relevant SK-MEL-28 melanoma mutant cell line, in which BRAF signaling is constitutively activated due to the substitution of valine for glutamic acid (V600E) (15). Significantly, this melanoma driver mutation is present in nearly 50% of all human melanomas (16, 17).

Given our prior results in breast cancer, this study was undertaken to assess the potential utility of CDDO-Me to alleviate immune suppression in the melanoma TME. Using this model system, we now show for the first time that T cells and CDDO-Me alter activation of human melanoma-conditioned macrophages. CDDO-Me inhibits CD16 surface expression on macrophages in tumor cell/macrophage co-cultures, while the addition of T cells to cultures results in decreased CD206, CD163, and CD16 expression. T cells also reduce CCL2, IL-6, and VEGF secretion from macrophages, and CDDO-Me augments this inhibition. We assess the requirement for T cell subsets in the attenuation macrophage immuno-suppression, and implicate CDDO-Me-mediated changes in STAT3 activation in the redirection of macrophage activation.

## Results

### Autologous T cells Enhance Attenuation of Melanoma-Conditioned Macrophage Markers

**Figure 1:**
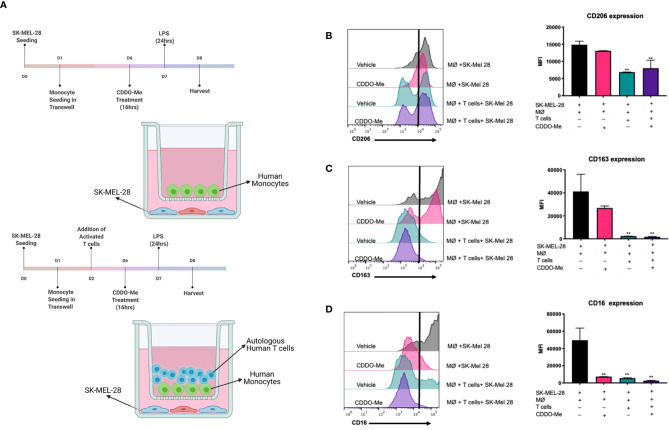
Surface expression of markers on melanoma-conditioned macrophages is attenuated by addition of autologous T cells. Human monocytes were incubated with 20 ng/ml M-CSF and cultured with SK-MEL-28 melanoma cells in the presence or absence of autologous T cells for 5 days. Cultures were then pre-treated with 300 nM CDDO-Me or DMSO (vehicle control) for 16 h, followed by LPS stimulation (10 ng/ml) for an additional 24 hrs. **(A)** Diagram of culture conditions. **(B)** CD206, **(C)** CD163, and **(D)** CD16 macrophage (MØ) surface marker expression levels were quantified using multi-color flow cytometric analysis and are presented in mean fluorescence intensity (MFI) units. Gating of positively stained cells was determined by single stained controls (negative stain control marked by black line). **p < 0.01 vs. co-culture untreated control (two-way ANOVA). Data shown are representative of results obtained from analysis of 3 individual donors and 3 separate experiments for each donor. Error bars represent standard deviation (SD).

In prior studies in an estrogen receptor negative (ER^-^) model of breast cancer, we demonstrated that CDDO-Me alters immune activation in the TME and redirects TAM activation from immunosuppressive to immunostimulatory (10, 11), To determine if CDDO-Me mediates similar effects in melanoma, human peripheral blood-derived monocytes were co-cultured for 7 days with SK-MEL-28 melanoma cells harboring the BRAF^V600E^ mutation using Transwells (**Figure 1A**). This culture system allows monocytes to differentiate *in vitro* in the presence of melanoma cell-secreted factors. Macrophage differentiation was verified by analysis of characteristic markers of activation (**Supplemental Figures 1** and **2**). The addition of CDDO-Me to cell cultures does not impair viability (**Supplemental Figure 6**).

To test if CDDO-Me alters immune activation of melanoma-conditioned macrophages, we first tested drug effects using a co-culture system. Cultures were pre-treated on Day 6 with 300 nM CDDO-Me or vehicle control for 16 hours (11), followed by activation with LPS for an additional 24 hours (**Figure 1A**). As demonstrated in **Figure 1C**, surface expression of CD16, a marker of non-classical monocytes (18), was inhibited by CDDO-Me, while expression of CD206 and CD163, which are upregulated on TAMs and correlate with poor patient prognosis (11, 19, 20), was not altered by drug treatment.

Because BRAF^V600E^ mutant tumors have been shown to regulate T cell recruitment during melanoma tumorigenesis (21), we next investigated the effect of CDDO-Me using a tri-culture that incorporate T cells to simulate a more complex tumor-like microenvironment (**Figure 1A**). Surprisingly, the addition of autologous T cells inhibited expression of CD206, CD163, and CD16 in tri-cultured melanoma-conditioned macrophages irrespective of CDDO-Me treatment (**Figures 1B–D**). Collectively, these results implicate a role for T cells in the regulation of pro-tumoral myeloid markers on macrophages in this human melanoma model system.

### Addition of Autologous Activated T Cells Augments CDDO-Me-Mediated Inhibition of IL-6 and CCL2

**Figure 2:**
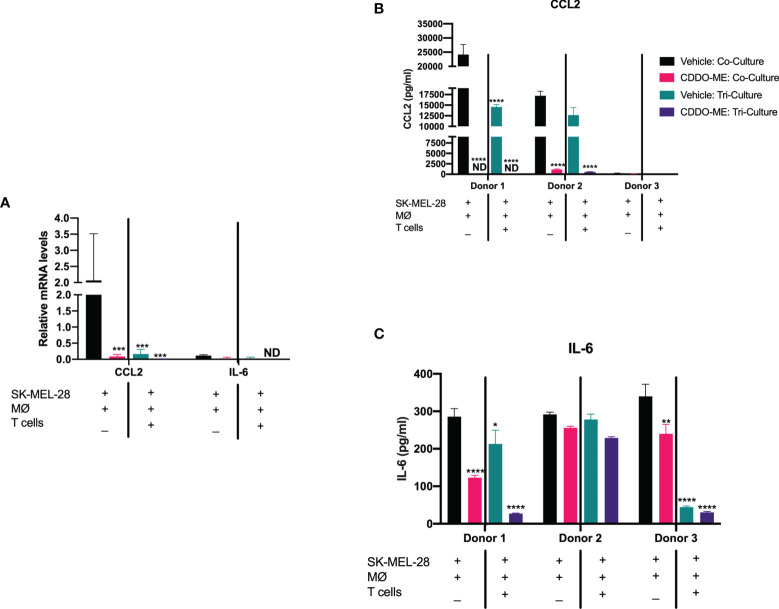
Addition of autologous activated T cells augments CDDO-Me effects on human melanoma-conditioned macrophage cytokine production. Human monocytes were incubated with 20 ng/ml M-CSF, autologous T cells and SK-MEL-28 melanoma cells for 5 days. Cultures were then pre-treated with 300 nM CDDO-Me or DMSO (vehicle control) for 16 h, followed by LPS stimulation (10 ng/ml) for an additional 24 hrs. Macrophages were immunophenotyped using flow cytometry to verify differentiation (**Supplemental Figure 1**). **(A)** Total RNA was extracted from LPS-activated co- or tri-cultures with or without CDDO-Me pretreatment. mRNA transcripts were measured by Taqman qRT-PCR. **(B)** ELISA analysis of CCL2 and **(C)** IL-6 collected from co- or tri-culture supernatants. *p < 0.05, **p < 0.01, ***p < 0.001, ****p < 0.0001 vs. untreated control (two-way ANOVA). Not detected (ND). Data shown are representative of results obtained from analysis of 3 individual donors. CDDO-Me effects on mRNA were analyzed by 3 separate experiments for each donor; 2 technical replicates were analyzed in each individual experiment. Error bars represent standard deviation (SD).

Given results obtained in **Figure 1** and prior studies demonstrating CDDO-Me-mediated changes in TAM cytokine production, we next investigated potential drug effects on CCL2 and IL-6 in co- and tri-cultures. As shown in **Figures 2A, B**, CDDO-Me treatment in the co-cultures significantly hampered CCL2 both at the mRNA as well as the protein levels. A similar trend was observed with IL-6 protein levels post CDDO-Me treatment. However, IL-6 mRNA levels were not significantly altered.

Strikingly, addition of autologous CD3^+^ T cells attenuated CCL2 mRNA expression (**Figure 2A**) and protein production of both IL-6 and CCL2 in vehicle-treated cultures as compared to the co-cultures, and this decrease in CCL2 and IL-6 levels was augmented by CDDO-Me (**Figures 2B, C**). These results suggest CDDO-Me attenuates immunosuppressive macrophage activation in BRAF^V600E^ mutant melanoma, consistent with prior observations in ER^-^ breast cancer (11, 22–24).

Monoculture CCL2 and IL-6 levels were measured to assess SK-MEL-28 vs. melanoma-conditioned macrophage protein production (**Supplemental Figure 3**). As previously reported, SK-MEL 28 produce little to undetectable levels of IL-6 and CCL2 (25), suggesting macrophages account for most of the observed IL-6 and CCL2 production.

### CDDO-Me Impairs Monocyte Migration in Co-Cultures

**Figure 3:**
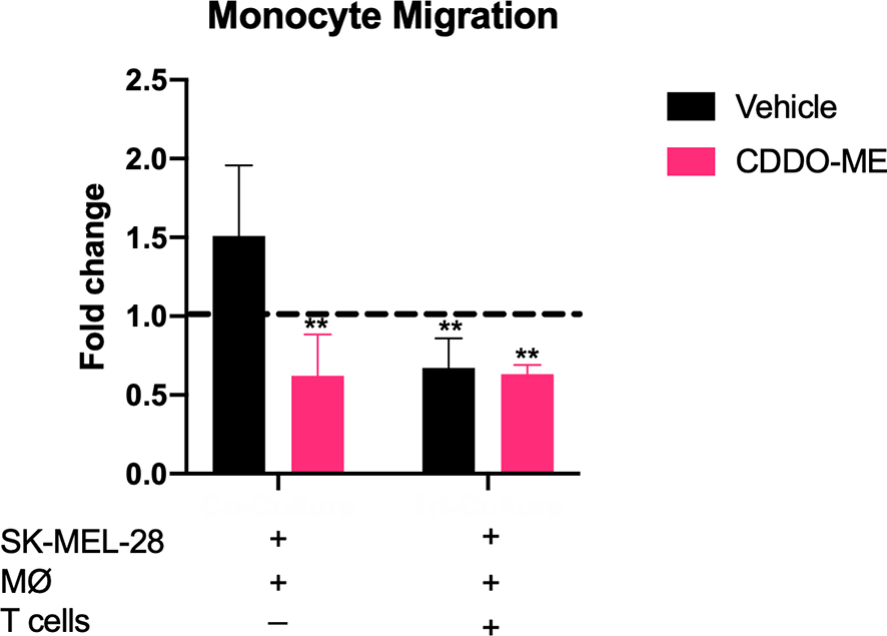
CDDO-Me impairs monocyte migration in co-cultures. Human monocytes were incubated for 8 h at 37°C in the upper portion of Transwells in 100 *μ*l of fresh media; feeder tray contained 150 *μ*l of undiluted or 1:20 diluted co or tri-culture supernatants. Relative monocyte migration towards co- or tri-cultured supernatants was measured. Media were collected from cultures that were maintained as described in **Figure 1**. Fold change is the ratio of sample fluorescence (minus background fluorescence) and internal control fluorescence (M-CSF-differentiated MØ supernatant). Control fold change of media (RPMI) alone is indicated by dotted black line. **p < 0.01 vs. untreated control (two-way ANOVA). Data shown are representative of results obtained from analysis of 3 donors. Error bars represent standard deviation (SD).

Because we showed that CDDO-Me inhibits TAM production of CCL2, which mediates myeloid cell recruitment to the TME (26–28), we hypothesized that CDDO-Me treatment would impair monocyte migration. We assessed the ability of CDDO-Me cultured media from co- and tri-cultures to impede migration. As expected, monocyte migration in co-cultured CDDO-Me media was significantly hindered (**Figure 3**). However, monocyte migration with tri-cultured media was hampered irrespective of CDDO-Me administration (**Figure 3**), consistent with results obtained in **Figure 2**. These results suggest CDDO-Me inhibits monocyte migration and this effect is enhanced by T cells.

### T Cell-Augmented CDDO-Me Attenuation of Melanoma-Conditioned Macrophage Activation Is Contact and T Cell Subset-Independent

**Figure 4:**
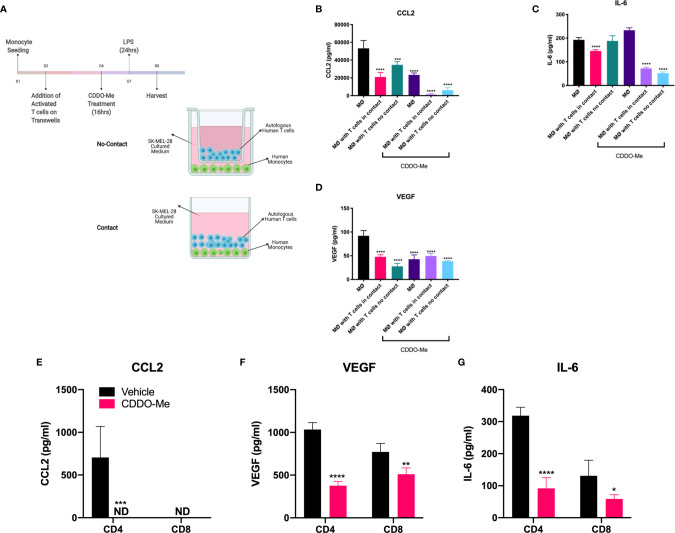
T cell-mediated inhibition of human melanoma-conditioned macrophage cytokine production is contact-and T cell subset-independent. Human monocytes were differentiated with 20 ng/ml M-CSF alone (MØ) or with M-CSF and conditioned media from SK-MEL-28 cells and autologous T cells for 5 days with contact (no Transwells) or without contact (separated by Transwells) as depicted in **(A)** Cultures were then pre-treated with 300 nM CDDO-Me or DMSO (vehicle control) for 16 h, washed, and activated with LPS (10 ng/ml) for an additional 24 hrs. Culture supernatants were collected and cytokine context assayed using ELISAs for CCL2, IL-6, and VEGF **(B–D)**. Panels **(E–G)** show results of LPS-stimulated tri-cultures consisting of either CD4 or CD8 T cells, SK-MEL-28 cells, and MØs pretreated with or without CDDO-Me. Significance was analyzed in **(B–D)** using two-way unpaired T-test (CDDO-Me-treated vs. vehicle) and in **(E–G)** using two-way ANOVA (tri-cultures vs. MØ untreated control). *p < 0.05, **p < 0.01, ***p < 0.001,****p < 0.0001; not detected (ND). Data are representative of results obtained from analysis of 3 separate experiments from 3 individual donors. Two technical replicates were analyzed in three separate experiments. Error bars represent standard deviation (SD).

To determine whether T cell effects on macrophage activation were mediated by soluble factors or if there were a requirement for direct contact between the cells, we performed Transwell co-culture studies. Monocytes were differentiated in conditioned media from SK-MEL-28 cells and incubated with autologous CD3^+^ T cells separated or not by Transwells (**Figure 4A**). As demonstrated in **Figures 4B, D**, production of CCL2 and VEGF was inhibited in a contact-independent manner in both vehicle and CDDO-Me-treated cultures. In contrast, direct T cell/macrophage contact was required for reduced IL-6 levels in vehicle-treated cultures, although this requirement was abrogated following CDDO-Me treatment (**Figure 4C**). These data suggest T cell-derived secreted factor(s) regulate macrophage cytokine production post-CDDO-Me treatment.

While TAMs are known to suppress T cell activation in the TME (29–31), the effect of activated T cells on TAM activation is poorly understood. Because the addition of T cells augmented CDDO-Me-mediated inhibition of macrophage production of IL-6 and CCL2, (**Figure 2**), we investigated the dependence of this synergy on T cell subset. We hypothesized that CD4^+^ T cells would be required for synergy with CDDO-Me, as CD4^+^ Th1 cells have been implicated in TAM redirection in B16 mouse melanoma models (7). Surprisingly, tri-cultures containing either CD4^+^ or CD8^+^ T cells were equally effective at suppressing production of CCL2 and IL-6 (**Figures 4E, G**) post-CDDO-Me treatment. Because TAMs sense hypoxia in avascular areas of tumors and react by producing pro-angiogenic factors such as VEGF-A (23, 24, 32), we interrogated the effect of CDDO-Me on VEGF-A production. As observed with CCL2 and IL-6, VEGF production was also hampered by both CD4^+^ and CD8^+^ T cell subsets post- CDDO-Me treatment (**Figure 4F**). Surface expression of CD206, CD163 and CD16 was not altered by either subset (**Supplemental Figure 4**). These results suggest CD4 and CD8 T cells inhibit immunosuppressive cytokine production in melanoma-conditioned macrophages, and that modulation of macrophage surface markers may require both T cell subsets, consistent with results in **Figure 1**.

### CDDO-Me Treatment Inhibits STAT3 Phosphorylation in Melanoma Cell Conditioned- Macrophages

**Figure 5:**
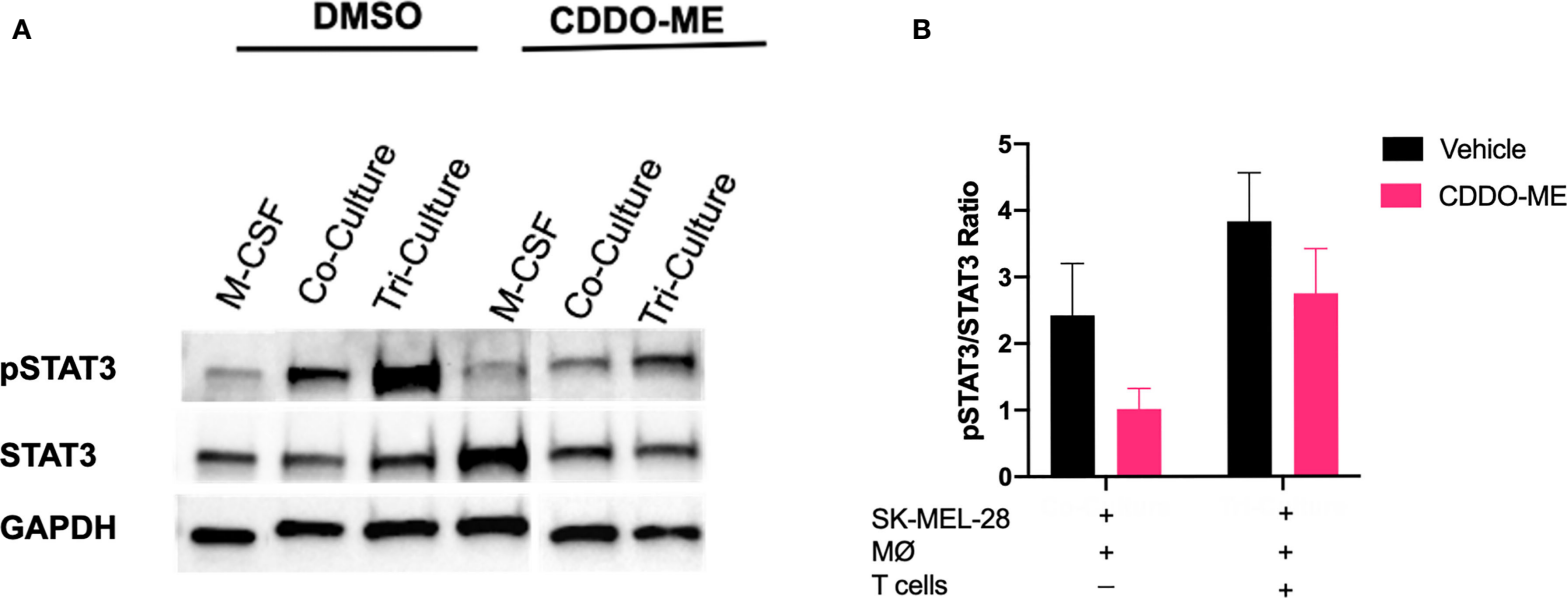
CDDO-Me treatment inhibits STAT3 phosphorylation in melanoma-conditioned macrophages. Whole cell lysates were prepared from human monocytes cultured for 5 days with cytokine in the absence (M-CSF) or presence of SK-MEL-28 cells (co-culture) or SK-MEL-28 cells and T cells (tri-culture). Cultures were treated with or without CDDO-Me (300 nM) for 16 h, washed, and then activated with LPS (10 ng/ml) for an additional 24 hrs. **(A)** Total and pSTAT3 levels were measured by immunoblot; GAPDH served as loading control. **(B)** Quantification of band intensity (ratio of pSTAT3/STAT3). Total STAT3 and pSTAT3 proteins were first normalized to their respective GAPDH signals. The ratio of GAPDH-normalized pSTAT3 to GAPDH-normalized total STAT3 was plotted for each treatment condition. vs. untreated control (two-way unpaired t test). Data are representative of results obtained from analysis of 3 individual donors and 3 separate experiments for each donor. Error bars represent standard deviation (SD).

CDDO-Me is a multifunctional drug that has been shown to inhibit activation of several signaling pathways important for cancer progression and metastasis, including MAPK, NF-κB, and STAT3 (10, 12, 33–37). Because STAT3 activation is associated with tumor growth, reduction of T cell infiltration, and maintenance of TAM immunosuppression in the TME, we hypothesized that one possible mechanism by which CDDO-Me attenuates TAM pro-tumoral activation is through inhibiting phosphorylation of STAT3 (11, 37–39). As a positive control for STAT3 phosphorylation, monocytes were differentiated with M-CSF alone in the absence of other cell types followed by stimulation with LPS. As demonstrated in **Figures 5A, B**, CDDO-Me reduced phosphorylation of STAT3 in all culture conditions, while total STAT3 levels were unaltered by CDDO-Me treatment. Full length immunoblots are provided in **Supplemental Figures 5A–C**. These results indicate that CDDO-Me inhibits STAT3 phosphorylation in human melanoma-conditioned macrophages and suggest CDDO-Me-mediated changes in myeloid activation may be due, at least in part, to suppression of STAT3 activation.

## Discussion

The high immunogenicity of melanoma tumors makes these malignancies an attractive target for immunotherapeutic treatment, as evidenced by the success of ipilimumab (1). However, many patients do not mount robust clinical responses to these and other targeted therapies, which is attributable, at least in part, to suppression of innate and adaptive immune responses by TAMs in the melanoma TME (2). Therefore, redirection of immunosuppressive myeloid cell activation may provide both a direct means of inhibiting melanoma growth and may enhance the efficacy of additional targeted and immuno-therapies.

In prior work, we showed that CDDO-Me redirects TAM activation in the breast TME from immunosuppressive to immunostimulatory (10, 11). To evaluate the potential efficacy of CDDO-Me in the relief of immune suppression in the human melanoma TME, we established a tri-culture model that incorporates human tumor cells, macrophages, and T cells. One advantage of this system is that it permits macrophage differentiation in a more physiologically-relevant context—i.e. in the presence of cells represented in the TME–instead of relying on differentiation with a single cytokine. In support of this, macrophages differentiated in co-and tri-culture express characteristic TAM surface markers and functionally resemble TAMs (**Figure 1** and **Supplemental Figure 1**). We now demonstrate that CDDO-Me and T cells inhibit surface expression of CD206, CD163 and CD16 and significantly attenuate production of CCL2, IL-6 and VEGF. Intriguingly, the addition of drug or T cells to cultures did not induce expression of CD80 or HLA-DR, which are associated with M1 macrophage activation (**Supplemental Figure 9**). This is in contrast to our previous work in breast cancer and suggests tissue-specific differences in the TME may account for these disparate effects. Nonetheless, these findings impute an important role to T cells in myeloid re-education in the context of CDDO-Me treatment and may provide insight as to the mechanisms by which CDDO-Me is able to effectively modulate the malignant melanoma TME.

In this regard, inhibited expression of CD206, which is upregulated on TAMs and is linked to tumor immunosuppression, angiogenesis, metastasis and relapse (19, 40), may function to restrict myeloid recruitment to the TME. Previous work has shown that CD206^+^ TAMs produce high levels of EGF (19), which induces CCL2 expression. Enhanced production of CCL2 results in increased recruitment of myeloid cells, which then differentiate into TAMs in the TME (41). Because the addition of T cells to melanoma cultures inhibits expression of CD206 (**Figure 1**), we hypothesize that EGF expression may also be limited, resulting in decreased CCL2 levels and inhibited myeloid chemotaxis to sites. Notably, CDDO-Me has been shown to inhibit both EGF and CCL2, consistent with our prior studies in breast cancer that reported decreased TAM numbers in tumors treated with CDDO-Me (10). *In vivo* studies are ongoing in our laboratory to investigate this mechanism.

Potential synergy between T cells and CDDO-Me may have significant therapeutic implications, given the observation that T cell-mediated reductions in CCL2 were enhanced by CDDO-Me treatment. CCL2 has been implicated in the early recruitment of monocytic-MDSCs, as well as regulatory CD4^+^ T cells and CCR2^+^ blood monocyte migration into tumors (21, 42), supporting an immunosuppressive TME. Given this, decreased CCL2 production mediated by T cells and CDDO-Me is likely a major contributor to the reduced monocyte migration we observed (**Figure 3**). This is of particular significance for patients with BRAF-positive melanoma treated with vemurafenib, as CCL2 and myeloid cell infiltration are hallmarks of vemurafenib resistance. Use of combination therapies, potentially including CDDO-Me, that target the CCL2/CCR2 axis may inhibit the development of treatment resistance in melanoma (43).

T cell and CDDO-Me-mediated changes in surface expression of CD163 may also result in changes in T cell recruitment to the TME. Prior work has shown that specific elimination of CD163^+^ TAMs increases T cell infiltration into TME, which is accompanied by inflammatory monocyte mobilization (44). Because our culture system already contains activated T cells, we hypothesize that the presence of these cells aids in the re-education of melanoma-conditioned macrophages and relieves immune suppression. This may be mediated in part through inhibited IL-6/JAK/STAT3 signaling (**Figures 2**, **4** and **5**).

Consistent with inhibited JAK/STAT3 signaling, IL-6 protein levels were reduced by the addition of T cells, and this effect was enhanced by CDDO-Me treatment. Attenuated IL-6 production may contribute to the inhibited STAT3 phosphorylation observed in **Figure 5**. STAT3 activation has been associated with tumor survival, growth and progression. Notably, phosphorylation of STAT3 in TAMs is induced by IL-6 signaling. This may lead to increases in pro-angiogenic VEGF, as well as increased transcription of IL-6, creating a positive feedback loop to further enhance JAK/STAT3 signaling (45).

Our work provides evidence for both T cell and CDDO-Me-mediated effects on melanoma cell-conditioned myeloid activation. CDDO-Me has shown efficacy in multiple tumor models and has been shown to regulate activation of multiple signaling pathways implicated in carcinogenesis and cancer progression, including Nrf2, NFκB, and STAT3. Because we have shown CDDO-Me-mediated attenuation of IL-6 production and STAT3 activation in melanoma-conditioned macrophages, we hypothesize that inhibition of JAK/STAT3 signaling is likely one of the mechanisms by which CDDO-Me inhibits tumor progression. In this regard, macrophage-derived IL-6 has been shown to induce defective CD4^+^ Th1 responses, leading to impaired antigen presentation and ineffective cytotoxic T cell priming (46). In addition, IL-6 blockade during CD40-mediated immune activation downregulates PD-1/PD-L1 on stimulated dendritic cells (46, 47). Although beyond the scope of the current study, we are interrogating additional mechanisms by which CDDO-Me potentiates decreased tumorigenesis and progression in ongoing studies in our lab.

Significantly, myeloid cell tumor infiltration and CCL2 levels are restored in acquired resistance to vemurafenib (48). As CDDO-Me impairs myeloid cell recruitment and attenuates CCL2 production, it is possible that the combination of CDDO-Me with vemurafenib may overcome the development of resistance. Additionally, vemurafenib alters the TME to support its combination with checkpoint inhibitors. Vemurafenib treatment has been shown to increase melanoma antigen expression and to increase antigen-specific T cell infiltration into melanoma tumors (49, 50). Therefore, CDDO-Me in combination with vemurafenib may facilitate the efficacy of checkpoint inhibition.

Our results showed that both CD4 and CD8 T cell subsets were equally capable of inhibiting melanoma-conditioned macrophage-derived CCL2, VEGF, and IL-6 in a contact-independent manner. This finding suggests secreted factor/s shared by both subsets may mediate this effect. One potential candidate is INF-γ, given its ability to polarize classically activated macrophages and to help with re-education of TAMs by CD4^+^ T cells (7). It is also possible that IL-2, which we have shown is induced by CDDO-Me in T cell monocultures and in tri-cultures that include monocytes, T cells, and melanoma cells (**Supplemental Figure 8**), contributes to T cell-mediated alterations in TAM activation. Notably, treatment of human and murine monocytes with IL-2 has been shown to attenuate immune suppression. Ongoing work in our lab is aimed at elucidating the role of IFN-γ, IL-2, and other potential T cell-derived mediators in the regulation of immunosuppressive macrophage activation.

Notably, while these studies were conducted with SK-MEL-28 cells, which express mutant BRAF (the V600E mutation), results obtained with other melanoma BRAF mutant or wild type cells may differ. This may limit broad extrapolation of CDDO-Me and T cell-mediated effects to melanoma generally. It will therefore be important in future studies to test additional melanoma cell lines using the culture system described in this work. Nevertheless, our results demonstrate for the first time both the potential utility of CDDO-Me and a role for T cells in the relief of immune suppression in human malignant melanoma.

Because immunosuppressive TAMs constitute a significant component of the melanoma TME, TAM re-education may alleviate the barrier for effective immunotherapeutic treatment of melanoma tumors. This study demonstrates that T cells and CDDO-Me attenuate immunosuppressive, pro-tumoral macrophage activation by inhibiting production of IL-6, VEGF and CCL2 and by reducing surface expression of CD206, CD163, and CD16. We further show that CDDO-Me attenuates STAT3 phosphorylation in melanoma-conditioned macrophages, and that T cells and CDDO-Me impair myeloid cell migration. These results implicate a role for T cells and CDDO-Me in the attenuation of immune suppression in the melanoma TME, and highlight their potential utility in enhancing the efficacy of other targeted immunotherapies.

## Methods

### Human Peripheral Blood Mononuclear Cell Isolation and Generation of Monocyte-Derived Macrophages

PBMCs were obtained by leukapheresis of healthy donors following written informed consent. Approval for this study was provided by the Institutional Review Board (IRB) of the Geisel School of Medicine, the Committee for the Protection of Human Subjects (protocol # 17011). This study was conducted in accordance with the human experimentation guidelines established by the Geisel School of Medicine Committee for the Protection of Human Subjects. Mononuclear cells were separated on Ficoll-Paque Premium (density: 1.077, GE Healthcare) and enriched for monocytes using cold aggregation. Monocyte purity was assessed at ≥95% using cytospin, Wright-Giemsa staining and flow cytometric analysis of CD14 (BioLegend, Cat.# 325618) expression.

Isolated CD14^+^ monocytes were differentiated with 20 ng/ml M-CSF in complete HEPES-buffered RPMI 1640 media supplemented with 10% FBS for 7 days. Macrophage polarization was verified using flow cytometry to measure expression of cell surface markers CD206 (clone: 15–2, Cat. #321103), CD163 (clone: GHI/61, Cat. #333606), and HLA-DR (clone: L243, Cat. #307618) and by qRT-PCR and ELISA analysis of secreted cytokines post-LPS stimulation.

### Co-and Tri-Cultures

Human monocytes were cultured in RPMI-1640 (Corning, Cat. #10-040-CV) supplemented with 10% FBS (HyClone, Cat. #SH30070.03), 0.25 M HEPES (Fisher Scientific, Cat. #BP299-100), and 1% penicillin streptomycin (Thermofisher, Cat. #15140122) and plated at 8 x 10^5^-1.2 x 10^6^/ml (tri- or co-culture, respectively) on day 1 in the top chamber of collagen-coated 0.4 μm PTFE membrane Transwells (Corning, Cat. #3491). Human malignant melanoma SK-MEL-28 cells carrying the BRAF^V600E^ mutation were cultured in E-MEM supplemented with 10% FBS and 1% penicillin streptomycin in the bottom chamber of Transwells at 2.5 x 10^4^/ml on day 0. For tri-culture studies, autologous T cells were activated for 2 days prior to addition to tri-cultures with anti-CD3/28/2 (Stemcell Technologies, Cat. #10990) from monocyte-depleted PBMCs in RPMI-1640 complete media supplemented with 10% FBS, 1% HEPES, 1% sodium pyruvate (HyClone, Cat. #SH30239.01), non-essential amino acids (HyClone, Cat. #SH30238.01), 0.055 mM BME (Gibco, Cat. #21985-023) and 1% penicillin streptomycin. T cells (2.5 x 10^5^/ml) were added to the top chamber of Transwells with monocytes. To compare the influence of CD4^+^ vs. CD8^+^ T cell subsets on tri-cultures, total CD3^+^ T cells were sorted using human CD4^+^ and CD8^+^ MicroBeads (Miltenyi Biotec, Cat. #130-045-101 and 130-045-201). For LPS activation studies, cells were pretreated with 300 nM CDDO-Me or DMSO vehicle control for 16 hours. After cultures were washed to remove drug, cells were stimulated with 10 ng/ml LPS (Sigma-Aldrich, Cat. #L4391-1MG) for an additional 24 hours. Graphic representations of culture assay systems are depicted in **Figures 1A** and **4A**.

### RNA Extraction, cDNA Synthesis, and Quantitative Real Time PCR

Total RNA was isolated using the quick RNA microprep kit (Zymo, Cat. #11-328M) per manufacturer’s instructions. Complementary DNA (cDNA) was synthesized from 100 ng total RNA and random hexamers using the qScript™ XLT cDNA SuperMix (QuantaBio, Cat. #955161-100). Quantitative real time PCR (qRT-PCR) was performed using TaqMan Probe single tube assays (Life Technologies, Cat. #4324018) for human CCL2 (Applied Biosystems, Cat. # Hs00234140), IL-6 (Cat. #Hs00985639_m1) and VEGF (Cat. # Hs00900055_m1) genes. The StepOnePlus Real-Time PCR System (Applied Biosystems) was used for amplification and detection. Threshold cycle number was determined using Opticon software. mRNA levels were normalized to β-actin, which control studies showed is not altered by CDDO-Me treatment (11), using the equation 2^-(Et-Rt)^, where Rt is the mean cycle threshold for the control gene and Et is the mean threshold for the experimental gene. Thermal cycling conditions for qRT-PCR consisted of an initial incubation at 50°C for 2 min and 95°C for 10 min, followed by 40 cycles of 95°C for 15 sec and 60°C for 1 min. Product accumulation was measured during the extension phase and all samples were run in triplicate.

### Enzyme-linked Immunosorbent Assay

Secreted protein expression in cell-culture supernatants was quantified by ELISA for CCL2 (Invitrogen, Cat. # 88-7391-88), IL-6 (Invitrogen, Cat. #88-7066-88), and VEGF (R&D Systems, Cat. #DY293B-05) according to manufacturers’ protocols.

### Flow Cytometry

All fluorophore-conjugated antibodies were obtained from BioLegend: anti-CD206-FITC (Cat. #321103), anti-CD163-PE(Cat. #333606), anti-CD64-PE/Cy7(Cat. # 305022), anti-CD80-APC (Cat. # 305220), anti-HLA-DR-APC/Cy7 (Cat. # 307618), anti-CD16-BV421 (Cat. # 360723), anti-CD1a-PerCP(Cat. # 300130), anti-CD45-APC/Cy7 (Cat. # 368516), anti-CD4-APC (Cat. # 300552), anti-CD8-BV510 (Cat. # 301048), anti-CD3-BV42 (Cat. # 344834). Cells were stained for 1 hour at 4°C with 2 mg/ml Globulins Cohn fraction II, III (Sigma) to inhibit non-specific antibody binding. In all conditions, doublets and multiplets were excluded by forward scatter pulse width (SSC-W) vs. forward scatter pulse area (SSC-A) gating. Gating of positively stained cells was determined by fluorescence-minus-one (FMO) controls. Cells were analyzed using an 8-color MACSQuant 10 (Miltenyi Biotec) with three laser sources (405 nm, 488 nm, 635 nm) and FlowJo 9.8.1 (Treestar).

### Immunoblot

Monocytes were differentiated with 20 ng/ml M-CSF alone or in co-culture with SK-MEL-28 cells or tri-culture with SK-MEL-28 cells and T cells as indicated, followed by treatment with 300 nM CDDO-Me or DMSO vehicle control for 16 hours as above. Cultures were then washed with media to remove drug and activated with 10 ng/ml LPS (Sigma-Aldrich, Cat. #L4391-1MG) for an additional 24 hours. Whole cell lysates were prepared using PRO-PREP™ Protein Extraction Solution (iNtRON Biotechnology, Cat. #17081). Lysates were analyzed for total protein concentration using a Micro BCA assay kit (Thermofisher Scientific, Cat. #23235). Four micrograms of each lysate were separated on Mini-PROTEAN TGS precast protein gels 4-15% (Bio-Rad, Cat. #45161084) and electrotransferred to nitrocellulose membrane (GE Healthcare, Cat. #10600012) in Tris-glycine buffer with 20% methanol. Membranes were blocked in 5% milk in 1xTBS and 0.05% Tween-20 (Santa Cruz, Cat. #SC-281695) for 1hr at RT. Blots were probed with primary detection antibodies for pSTAT3 (Cell Signaling, Cat. #4113S), STAT3 (Cell Signaling, Cat. #4904S) and GAPDH (Abclonal, Cat. #AC002). Primary antibodies for pSTAT3 and STAT3 were incubated at a 1:2000 dilution in Tris-buffered saline with 0.05% Tween 20 (Santa Cruz) and 5% BSA (Fischer BioReagents, Cat. #BP1600100) for 1hr at 4°C with rotation. Primary antibody for GAPDH was incubated at a 1:10000 dilution in Tris-buffered saline with 0.05% Tween 20 and 5% milk (Nestle Carnation) for 1hr at 4°C with rotation. Following incubation with primary antibodies, blots were incubated with horseradish peroxidase-conjugated secondary antibodies (goat anti-mouse or goat anti-rabbit) (Bio-Rad, Cat. #170-6516 or #170-6515) at a 1:2,000 dilution in Tris-buffered saline with Tween 20 (TBST) and 5% milk for 1hr at room temperature with gentle agitation on a rocker, followed by thorough washing in TBST. Blots for STAT3 and GAPDH were visualized using Pierce™ ECL Western Blotting Substrate (Thermofisher Scientific). Blots for pSTAT3 were visualized using SuperSignal™ West Femto Maximum Sensitivity Substrate (Thermo Scientific). To control for loading errors, total STAT3 and pSTAT3 proteins were normalized to their respective GAPDH signals. Using these normalized values, the ratio of pSTAT3 to total STAT3 was plotted for each co-culture and tri-culture condition.

### Migration Assay

Migration assays were performed using freshly-isolated monocytes in a modified Boyden chamber-based system. HTS Transwells, 96-well (5.0 *μ*m pore, Corning, Cat. #3388), were used to assay chemotaxis. 150*μ*l of tri-culture supernatant or media with and without CCL2 (Peprotech, Cat. #300-04-5ug) control (1 ng/ml) were transferred to the 96-well feeder tray (bottom). A cell migration chamber plate with 5.0 *μ*m pores was placed over the 96-well feeder tray. A volume of 100*μ*l containing 200,000 fresh monocytes cells/well resuspended in complete RPMI was transferred to each inner well of the cell migration chamber plate. The 96-well migration plate was placed in a CO_2_ incubator at 37°C for 8 hours. At the conclusion of the incubation, migrated cells were lysed and dyed with CyQuant (Invitrogen, Cat. #C7026) GR, then read on a fluorescence plate reader at Excitation 480nm/Emission 520nm.

### Statistical Analysis

Figures are representative of at least three independent experiments as indicated in Figure Legends. All experiments were repeated at least three times, unless otherwise noted, and at least 2 technical replicates of each analyte were included in each assay. Results are described as mean ± SD and were analyzed by unpaired student’s t-Test or two-way ANOVA for multiple comparison (as indicated in Figure Legends) using GraphPad Prism 8. Significance was achieved at p < 0.05.

## Data Availability Statement

The raw data supporting the conclusions of this article will be made available by the authors, without undue reservation.

## Ethics Statement

PBMCs were obtained by leukapheresis of healthy donors following written informed consent. Approval for this study was provided by the Institutional Review Board (IRB) of the Geisel School of Medicine, the Committee for the Protection of Human Subjects (protocol 17011). This study was conducted in accordance with the human experimentation guidelines established by the Geisel School of Medicine Committee for the Protection of Human Subjects. The patients/participants provided their written informed consent to participate in this study.

## Author Contributions

GT, KL, and PP wrote the main text of the manuscript. GT, HY, CP, PS, NK, and RB prepared **Figures 1**–**5** and **Supplemental Figures**. All authors contributed to the article and approved the submitted version.

## Funding

This study was supported by Norris Cotton Cancer Center Developmental Funds (Prouty Pilot Project to PP) and The National Cancer Institute (NCI) of the National Institutes of Health under award number R01CA226690 (to KL). GT is supported by the American Association of Immunologists Careers in Immunology Fellowship, and RB is supported by the John Osborn Polak Endowment. The funders had no role in study design, data collection and analysis, decision to publish, or preparation of the manuscript.

## Conflict of Interest

KL is an inventor of patents dealing with chemical synthesis of new triterpenoids and their application in treatment of cancer, as well as in inflammatory diseases, including human kidney disease.

The remaining authors declare that the research was conducted in the absence of any commercial or financial relationships that could be construed as a potential conflict of interest.

## Publisher’s Note

All claims expressed in this article are solely those of the authors and do not necessarily represent those of their affiliated organizations, or those of the publisher, the editors and the reviewers. Any product that may be evaluated in this article, or claim that may be made by its manufacturer, is not guaranteed or endorsed by the publisher.
