# Supplementary material for: T Cells and CDDO-Me Attenuate Immunosuppressive Activation of Human Melanoma-Conditioned Macrophages
Source: Front Immunol. 2022 Feb 21;13:768753. doi: 10.3389/fimmu.2022.768753 (PMC8898828; doi:10.3389/fimmu.2022.768753)
Supplement: Supplementary file 1 [file DataSheet_1.docx]

**Supplemental Figure 1:** **Phenotype of macrophages mono- or co-cultured with SK-MEL-28 cells.** Human monocytes were incubated with 20 ng/ml M-CSF and cultured with or without SK-MEL-28 melanoma cells for 6 days followed by LPS stimulation (10 ng/ml) for an additional 24 hrs. CD206, CD163, and CD16 TAM surface marker expression levels were quantified using multi-color flow cytometric analysis and are presented in mean fluorescence intensity (MFI) units. Gating of positively stained cells was determined by single stained controls (negative stain control marked by black line). *p<0.05, **p<0.01, ***p<0.001 vs. mono-culture (two-way ANOVA). Data are representative of results obtained from three separate experiments performed with cells from three distinct donors. Error bars represent standard deviation (SD).

**Supplemental Figure 2: Protein secretion of macrophages (MØs) mono- or co-cultured with SK-MEL-28 cells.** Human monocytes were incubated with 20 ng/ml M-CSF and cultured with or without SK-MEL-28 melanoma cells for 6 days followed by LPS stimulation (10 ng/ml) for an additional 24 hrs. Macrophages were immunophenotyped using flow cytometry to verify differentiation (Supplemental Figure 1). Secreted levels of CCL2, VEGF and IL-6 were measured using ELISA. *p<0.05, **p<0.01, ***p<0.001 vs. mono-culture control (two-way ANOVA). Not detected (ND). Data are representative of results obtained from experiments with cells from three distinct donors. Error bars represent standard deviation (SD).

**Supplemental Figure 3: Cytokine secretion in monocultures.** Human monocytes or SK-MEL-28 cells were incubated with 20 ng/ml M-CSF for 6 days. Cultures were then pre-treated with 300 nM CDDO-Me or DMSO (vehicle control) for 16 hrs, followed by LPS stimulation (10 ng/ml) for an additional 24 hrs. Secreted protein levels of CCL2 and IL-6 were quantified by ELISA. *p<0.05, **p<0.01, ***p<0.001 vs. vehicle control (two-tailed T-test). Not detected (ND). Data are representative of results obtained from experiments with cells from three distinct donors. Error bars represent standard deviation (SD).

**Supplemental Figure 4**: **Surface expression of TAM markers in CD4^+^ or CD8^+^ tri-cultures.** Human monocytes were incubated with 20 ng/ml M-CSF and cultured with SK-MEL-28 melanoma cells in the presence or absence of autologous CD4^+^ or CD8^+^ T cells for 5 days. Cultures were then pre-treated with 300 nM CDDO-Me or DMSO (vehicle control) for 16 hrs, followed by LPS stimulation (10 ng/ml) for an additional 24 hrs. (*A)* CD4^+^ tri-cultures. (*B)* CD8^+^ tri-cultures. Surface marker expression (CD206, CD163 and CD16) levels were quantified using multi-color flow cytometric analysis and are presented in mean fluorescence intensity (MFI) units. Gating of positively-stained cells was determined by single stained controls (negative stain control marked by black dotted line). *p<0.05, **p<0.01, ***p<0.001 vs. co-culture untreated control (two-way ANOVA). Data are representative of results obtained from three separate experiments using cells from three distinct donors. Error bars represent standard deviation (SD).

**Supplemental Figure 5**: **Full-length western blot of mono, co, and tri-cultured macrophages treated with or without CDDO-Me.** Whole cell lysates were prepared from human monocytes differentiated with cytokine alone (20 ng/ml M-CSF or GM-CSF) or in co- or tri-cultures for 5 days, followed by treatment with or without CDDO-Me (300 nM) for 16 hrs and 10 ng/ml LPS for an additional 24 hrs. *(A)* GAPDH*, (B)* STAT3 and *(C )*pSTAT3. Precision Plus Protein Dual Color Standard is indicated in lane #1 of each blot to determine relative molecular weights.

**Supplemental Figure 6: Monocyte, macrophage, T cell and SK-MEL-28 viability post-CDDO-Me treatment.** *(A)* Human monocytes were cultured overnight; (*B)* macrophages were differentiated for 3 days with 20 ng/ml M-CSF; *(C)* T cells were activated *in vitro* with anti-CD3/28/2 antibody for 2 days; and *(D)* SK-MEL-28 cells were cultured until confluent. Cells in panels A-D were then treated with 300 nM CDDO-Me or DMSO (vehicle control) for 16 hrs. Cell viability post-treatment was analyzed by multicolor flow cytometry using the Fixable Near IR Dead cell stain kit. Gating of positively stained cells was determined by single stained controls. *p<0.05, **p<0.01, ***p<0.001 vs. co-culture untreated control (two-way ANOVA). Data are representative of results obtained from three biological replicates. Error bars represent standard deviation (SD).

**Supplemental Figure 7: Cytokine secretion in monocultures with or without *in vitro-*activated T cells.** *(A)* Human T cells were activated or not with anti-CD3/28/2 for 2 days and subsequently cultured for 3 additional days. Cultures were then pre-treated with CDDO-Me for 16 hrs, followed by LPS stimulation (10 ng/ml) for an additional 24 hrs *(B)* Human T cells were activated with anti-CD3/28/2 for 2 days and cultured for 3 additional days. Cells were then pre-treated with 300nM CDDO-Me or DMSO (vehicle control) for 16 hrs, followed by LPS stimulation (10 ng/ml) for an additional 24 hrs. Secreted protein levels of CCL2, VEGF, IL-10 and IL-6 were quantified by ELISA. *p<0.05, **p<0.01, ***p<0.001 vs. activated control or vehicle control (two-way ANOVA). Not detected (ND). Data are representative of results obtained using cells from two distinct donors. Two technical replicates were analyzed in each individual experiment. Error bars represent standard deviation (SD).


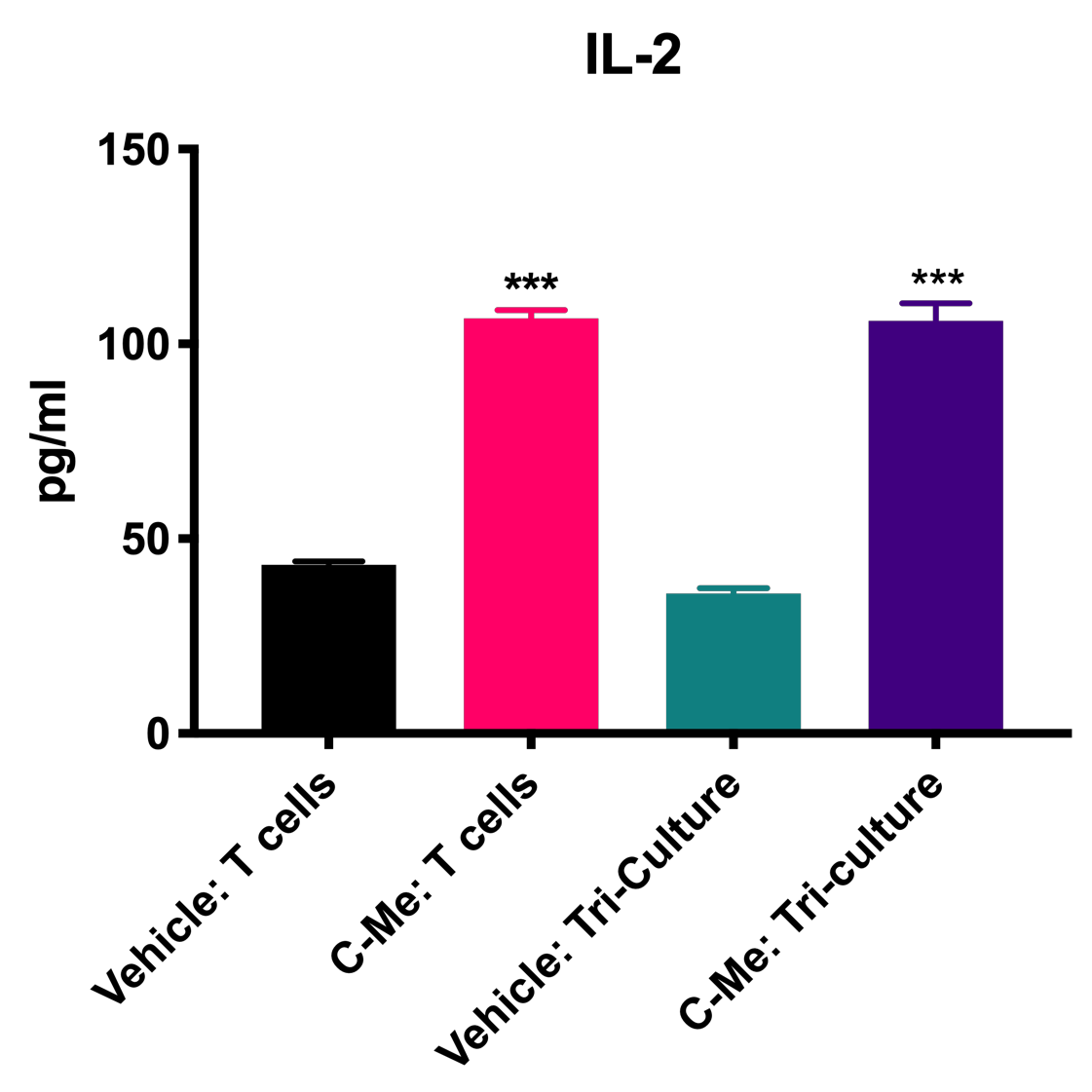


**Supplemental Figure 8: IL-2 secretion in mono- and tri-cultures with *in vitro-*activated T cells.** Human T cells were incubated with 20 ng/ml M-CSF and cultured with or without SK-MEL-28 melanoma cells in the presence or absence of autologous monocytes for 5 days. Cultures were then pre-treated with 300 nM CDDO-Me or DMSO (vehicle control) for 16 hrs, followed by LPS stimulation (10 ng/ml) for an additional 24 hrs. Secreted protein levels of IL-2 were quantified by ELISA. *p<0.05, **p<0.01, ***p<0.001 vs. vehicle monoculture control (two-way ANOVA).

**Supplemental Figure 9:**  **M1 macrophage surface marker expression on human melanoma-conditioned macrophages is not significantly altered by CDDO-Me treatment.** Human monocytes were incubated with 20 ng/ml M-CSF and cultured with SK-MEL-28 melanoma cells in the presence or absence of autologous T cells for 5 days. Cultures were then pre-treated with 300 nM CDDO-Me or DMSO (vehicle control) for 16 hrs followed by LPS stimulation (10 ng/ml) for an additional 24 hrs. (*A*) HLA-DR and (*B*) CD80 TAM surface marker expression levels were quantified using multi-color flow cytometric analysis and are presented in mean fluorescence intensity (MFI) units. Gating of positively stained cells was determined by single stained controls. *p<0.05, **p<0.01, ***p<0.001 vs. co-culture untreated control (two-way ANOVA). Data shown are representative of results obtained from analysis of 3 individual donors and 3 separate experiments for each donor. Error bars represent standard deviation (SD).
